# Global trends in the research on benign paroxysmal positional vertigo: A 20-year bibliometric and visualization analysis

**DOI:** 10.3389/fneur.2022.1046257

**Published:** 2022-10-17

**Authors:** Fangwei Zhou, Bingxi Yu, Jiali Luo, Yifei Ma, Jianyao Li, Tian Zhang, Guodong Yu

**Affiliations:** ^1^Department of Otorhinolaryngology Head and Neck Surgery, Affiliated Hospital of Guizhou Medical University, Guiyang, China; ^2^Department of Otolaryngology, Xingyi People's Hospital, Xingyi, China; ^3^Department of Development and Planning, Guizhou Medical University, Guiyang, China

**Keywords:** benign paroxysmal positional vertigo, bibliometrics, hotspots, visualization analysis, global trend

## Abstract

**Background:**

Benign paroxysmal positional vertigo (BPPV) is the most common cause of peripheral vestibular vertigo. Although BPPV is benign, its underlying mechanisms are complicated, and patients diagnosed with BPPV are significantly affected by it in their daily lives. Hence, this study's purpose was to investigate global trends and frontiers in the field of BPPV.

**Methods:**

We searched the research literature published from 2002 to 2021 on BPPV using two databases from the Web of Science Core Collection, and we conducted a bibliometric and visualization analysis. Bibliometric tools were used to perform co-authorship, co-citation, and co-occurrence analyses of countries or regions, institutions, authors, journals, keywords, and references.

**Results:**

In all, 1,419 publications from 4,594 authors, 1,542 institutions, and 65 countries or regions with 71 subject categories were included in the study. The number of articles increased gradually from 2002 to 2021. Seoul National University, the University of Munich, and Osaka University were among the leading institutions with the most publications, while United States of America, South Korea, and China were the leading countries. JS Kim was the most prolific author, Otology & Neurotology was the most prolific journal, and Otorhinolaryngology was the most published subject category. The five most frequently occurring keywords were BPPV, vertigo, dizziness, nystagmus, and management and the top research hot spots were osteoporosis and vitamin D.

**Conclusion:**

This study systematically analyzed trends in global scientific research on BPPV. The academic understanding of BPPV has improved significantly over the last two decades, with osteoporosis and vitamin D the two main research hot spots in the field of BPPV in recent years. These findings provide direction for current research to grasp the trends and research frontiers of current research.

## Introduction

Benign paroxysmal positional vertigo (BPPV) is the most common peripheral vestibular condition observed in neurology outpatient departments, accounting for ~20–30% of all vestibular complaints (1). Characteristics of BPPV include recurrent transient vertigo with nystagmus when lying down or getting up, extending or turning the neck or turning over in bed (1, 2). The pathologic mechanism of BPPV is based on dislodged otoliths leaving the utricle and freely floating in the semicircular canals or attached to the cupula, making the labyrinth sensitive to gravity (2). In the majority of situations, BPPV is idiopathic, but it can also be secondary (after otologic surgery, Meniere's disease, head trauma, migraine, virus infection, and a prolonged recumbent position (3). As one of the most common causes of dizziness and vertigo worldwide, BPPV accounts for 24.1% of all hospital visits for dizziness and vertigo, with an overall prevalence of 2.4% (4, 5). BPPV is the most prevalent disease among older females, with the highest prevalence among those ~60 years of age, with a male to female ratio of 2.4:1 (6). Recurrences of BPPV are frequent, ranging from 15 to 20% per year (7).

Although it is not malignant, BPPV can significantly limit patients' daily activities (2, 6). The medical cost of diagnosing BPPV is ~$2,000 (USD) in the United States of America (USA), $450 in Spain, $600 in China, and $180 in South Korea (7). The annual healthcare burden due to BPPV in the USA has been estimated to be ~$2 billion, and this burden is likely to grow with the aging of the population (6). A previous study found that the number of hospital visits for dizziness and vertigo per 1,00,000 general population was ~3,974 in 2019 and is likely to be 6,057 in 2050, which will be a 52% increase (5). Although canalicular repositioning is an effective therapy, the recurrence rate of BPPV is reported to be ~50% at the 10-year follow-up (6, 8). Frequent recurrences may result in significant disruptions in the daily activities of patients with BPPV. The etiology of BPPV is mostly unclear and may be related to head trauma, prolonged bed rest, or various diseases related to the inner ear (6). Many studies have revealed that BPPV might be related to other comorbid diseases, such as diabetes, hypertension, hyperlipidemia, thyroid disorders, and osteoporosis, which may contribute to its increased recurrence rates after treatment (9). Currently, there are few reports synthesizing the diverse information on BPPV, and such reports could provide scholars with a comprehensive visualization of the research trends and frontier hot spots regarding BPPV.

Bibliometrics is a method that has gradually developed in recent years to analyze the existing scientific research literature (10). Traditional reviews cannot clearly reveal the networks, structures, interactions, intersections, or the evolution of various pathophysiological and molecular biological mechanisms (11). These relationships generate new knowledge, but they might not identify hot spots for analyses or indicate which frontiers should be explored. The dynamic character of the rapidly evolving international literature poses a challenge to strategies for mining literature. Bibliometric analysis can compensate for the many limitations of traditional literature reviews by providing a macroscopic point of view of the scholarly literature and identifying the frontiers of research. Bibliometrics focuses on the analysis of bibliographic and content information of a specified field through quantitative analysis and statistical methods. It is used to analyze publications in various fields in order to assess the quality of the publications and trends over a certain period of time (12). Through scientific identification, comprehensive statistics, and systematic sorting by researchers, a large amount of literature in a field can be quickly interpreted, and key information can be extracted.

Over the past two decades, research worldwide has revealed the mechanisms of BPPV and its treatments. However, no bibliometric analyses of trends in global research on BPPV have been published. Hence, the aim of this research was to explore the trends in and hot spots of BPPV research worldwide in databases, which include clinical studies, basic experimental studies, and systematic reviews between 2002 and 2021. This study set out to identify prolific authors and their cooperative partnerships, countries/regions, and institutions, and a network analysis of keywords to reflect the development of trends and research frontiers. The results of this bibliometric analysis will assist clinicians and researchers to develop an understanding of the framework of knowledge and issues in the area, offer directions and ideas for research topics, and help improve the quality of publications in the field.

## Materials and methods

### Data acquisition and search strategy

**Figure 1:**
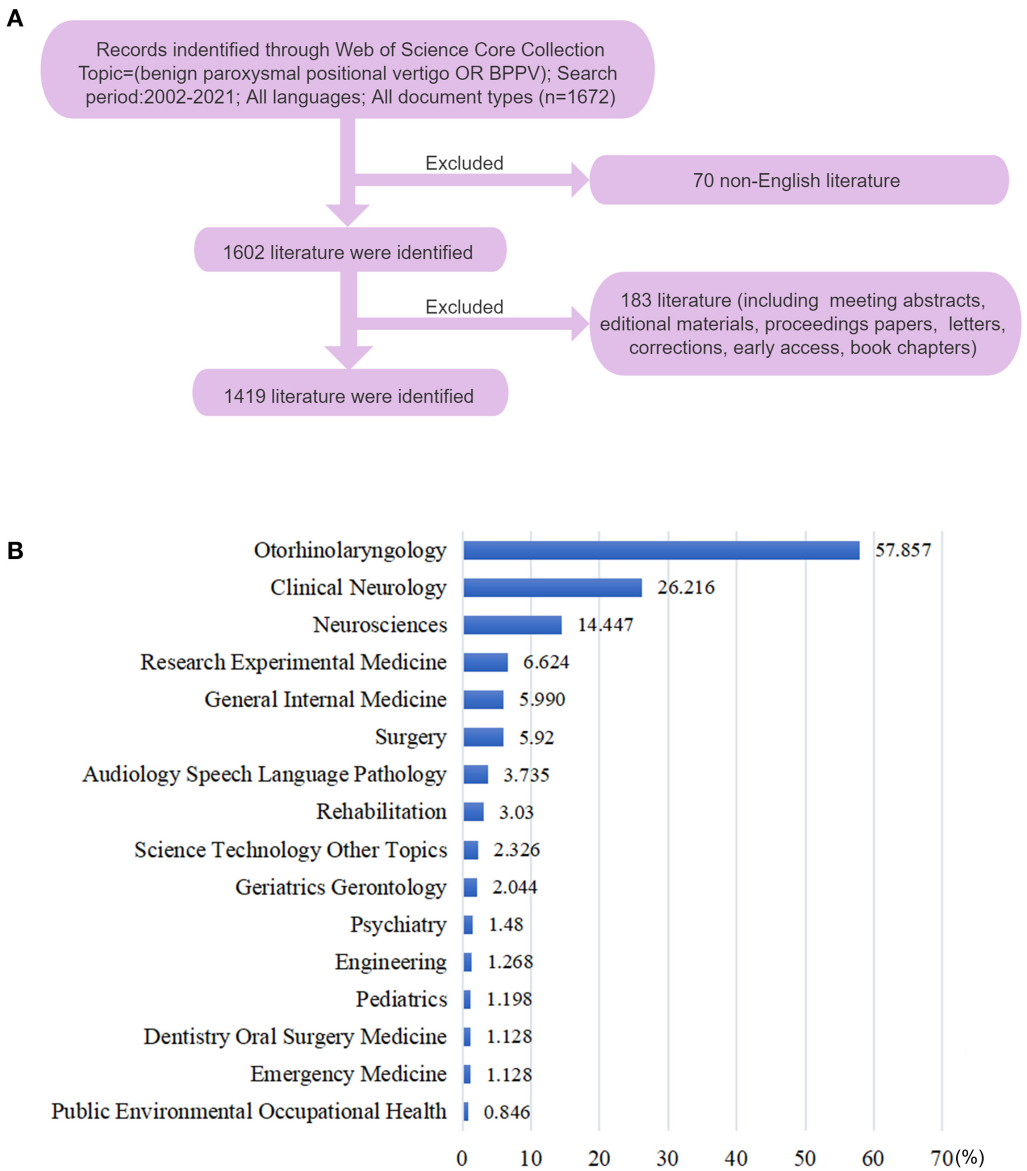
**(A)** Flow diagram of the publication selection process for a bibliometric analysis. **(B)** Subject categories of the publications.

This bibliometric study was conducted using the Science Citation Index Expanded and Social Sciences Citation Index databases from the Web of Science Core Collection. The search formula was as follows: Topics = (“benign paroxysmal positional vertigo” OR “BPPV”). The time span for the study was set to 2002–2021. Only articles and reviews in English were searched to ensure the retrieved publications were representative of those reporting global trends in the field of BPPV. All of the records and cited references in the search results were downloaded in a plain-text and tab-delimited file formats. The selection of the publications is depicted in Figure 1A. Retrieval and downloads of the documents were completed within one day (June 1, 2022) to avoid bias due to routine updates of the database. Two researchers independently examined these data, which were secondary data from the database and without any individual's personal information. Therefore, this study does not require informed consent.

### Bibliometric analysis

All of the publications downloaded from the database were transferred to VOSviewer 1.6.16, CiteSpace 5.8. R3, the bibliometric software package of R-Studio 3.2.1^1^, and an online tool for bibliometric analysis^2^ to conduct the visualization analysis. The characteristics of these publications, including their countries, institutions, authors, journals, keywords, and references were described. The impact factor and category quartile of the journals were derived from Journal Citation Reports, 2021. This study's researchers inquired about the H-index of several indicators, which is considered a crucial measure of the scientific value of the study. Microsoft Excel 2020 was used to create tables and present publishing trends among the global publications.

CiteSpace is a Java-based visualization application of bibliometric analysis used for building network maps (13). It was used in this study to calculate centralities and create a collaborative network visualization graph for institutions and yearly visualizations of citation bursts of keywords and references. Visual network graphs consisted of numerous nodes and links. The size of each node represented the frequency of an item's occurrence. High centrality nodes with purple rings indicated a turning point or pivot point in the domain. Centrality was used to evaluate the importance of a node in a network; as the centrality increases, so does the number of links through the node (14, 15). To identify research frontiers, bursts were used as indicators to detect increases in the rate of citations of keywords. In this study, co-citation analysis of references was conducted to construct a relevant knowledge graph, and the detection of keyword bursts was conducted to examine recurring keywords in each period.

VOSviewer, is a novel scientometric tool used to create maps from databases and to visualize the data for analysis (16). In the network visualization diagram, different clusters are represented by different colors, and the lines among the circles represent cooperative relationships. In the visualization chart with the year of publication, the shades of color refer to different times. A correlation network diagram was created, in which circles represent elements, such as country, institution, author, and keyword, and the size of the circle represents the frequency or number, with larger circles indicating more publications or occurrences; links among the circles reflect co-authorship, co-occurrence, or co-citation relationships.

In this research, we used VOSviewer to identify keywords and authors. The network diagrams in this study were built according to the co-occurrence of keywords and the collaborative relationships of authors. To identify important terms in the study, keywords that appeared more than 10 times were included in the keyword co-occurrence network for analysis. VOSviewer classified keywords into different clusters according to subject categories, with different colors representing different clusters.

## Results

### Analysis of publications output

**Figure 2:**
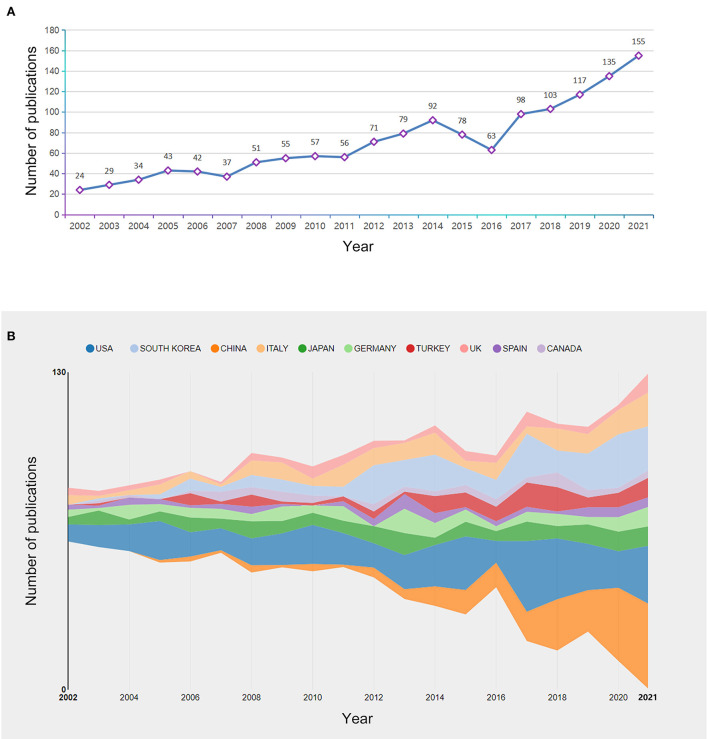
**(A)** Yearly output of publications from 2002 to 2021. **(B)** Upward trend in publishing BPPV related research from 2002 to 2021 among the top-10 countries/regions with the most publications.

Our search yielded 1,419 eligible publications (including 1,295 original articles and 124 review articles) on BPPV research between 2002 and 2021. Each publication was matched with at least one subject category in the Web of Science Core Collection. The 1,419 publications on BPPV were categorized into 71 subject categories (Figure 1B). Otorhinolaryngology accounted for the most publications (821), followed by Clinical Neurology (372), Neurosciences (205), Experimental Research Medicine (94), and General Internal Medicine (85). An upward trend in the number of publications distributed by year, is shown in Figure 2A. Notably, research activities in BPPV increased dramatically between 2017 and 2021, with 608 articles published over this five-year period, which represented > 40% of the total number of related articles.

### Distribution by countries and institutions

**Figure 3:**
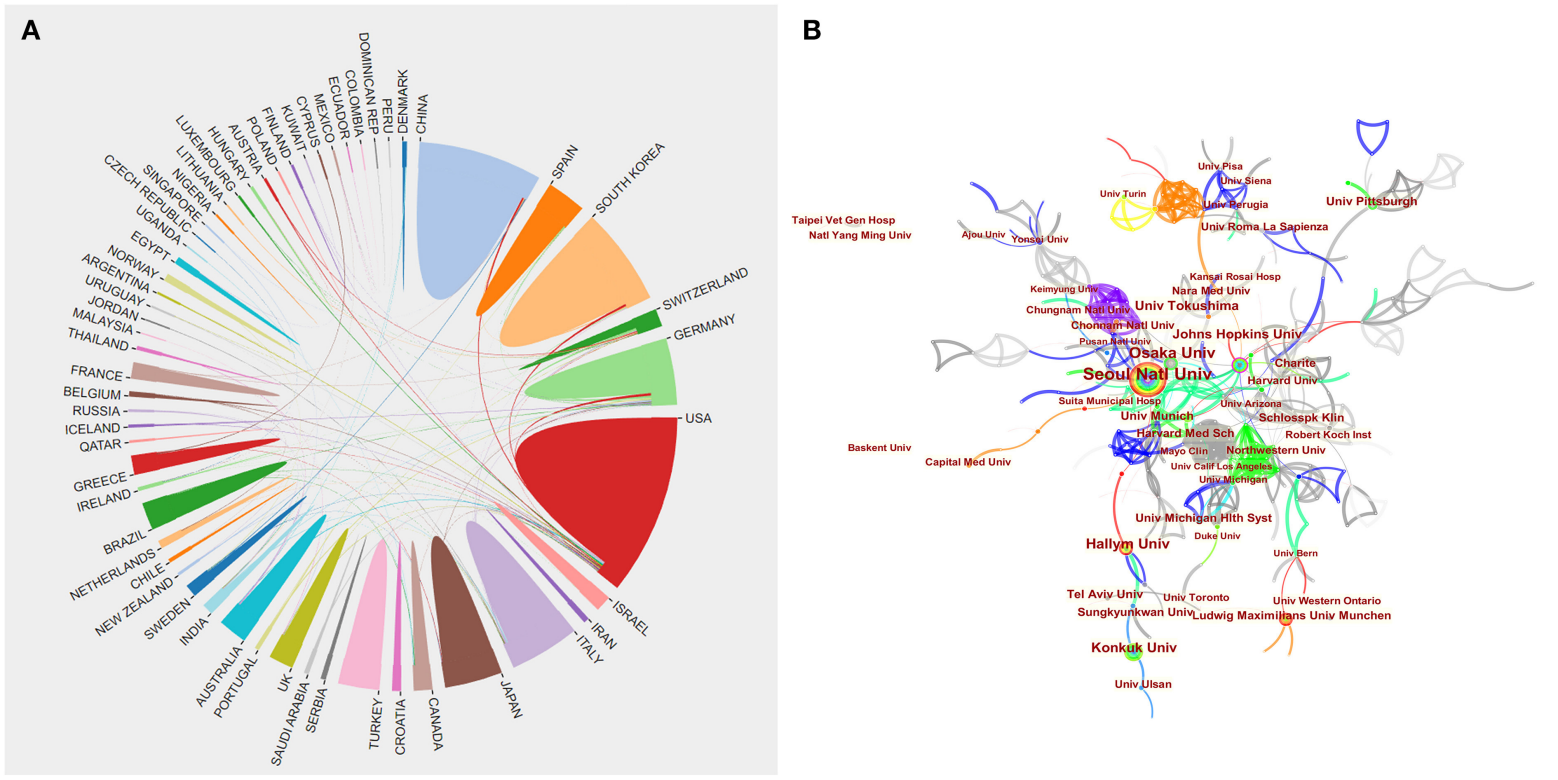
**(A)** A network map of the cooperative relationship between countries or regions. **(B)** A visual map of institutions' contributions to BPPV research publications.

In all, 1,419 papers from 65 countries and 1,542 institutions were published. Tables 1,2 indicate the countries and institutions with the most publications on the topic. The USA was the country with the most publications (301), followed by South Korea (169), China (139), Italy (119), and Japan (113) (Table 1). Figure 2B shows an upward trend in publishing among the top-10 countries with the most publications. Figure 3A maps the cooperating networks across countries; the USA had the highest centrality score (0.59), followed by Germany (0.21), Canada (0.15), Japan (0.07), and the United Kingdom (0.06). The CiteSpace analysis of the distribution of institutions contributing to the field (Figure 3B) revealed the five most productive institutions: Seoul National University (49), University of Munich (40), Osaka University (33), Johns Hopkins University (29), and Harvard University (28). The top-3 institutions in terms of centrality were all located in the USA: Johns Hopkins University (0.12), University of Michigan (0.08), and Harvard University (0.07).

**Table 1:** The top-10 countries with the most publications in the field of BPPV.

**Rank**	**Country**	**Count**	**Centrality**
1	USA	301	0.34
2	South Korea	169	0.00
3	China	139	0.01
4	Italy	119	0.02
5	Japan	113	0.07
6	Germany	91	0.21
7	Turkey	82	0.00
8	UK	56	0.06
9	Spain	49	0.00
10	Canada	47	0.15

BPPV, benign paroxysmal positional vertigo; USA, United States of America; UK, United Kingdom.

**Table 2:** The top-10 institutions with the most publications on BPPV.

**Rank**	**Institution**	**Count**	**Country**	**Centrality**
1	Seoul National University	49	South Korea	0.04
2	University of Munich	40	Germany	0.04
3	Osaka University	33	Japan	0.03
4	Johns Hopkins University	29	USA	0.12
5	Harvard University	28	USA	0.07
6	Hallym University	25	South Korea	0.01
7	League of European Research Universities	25	-	0.02
8	University of Michigan	23	USA	0.08
9	Konkuk University	22	South Korea	0.00
10	Tokushima University	22	Japan	0.00

BPPV, benign paroxysmal positional vertigo; USA, United States of America.

### Distribution by authors

**Figure 4:**
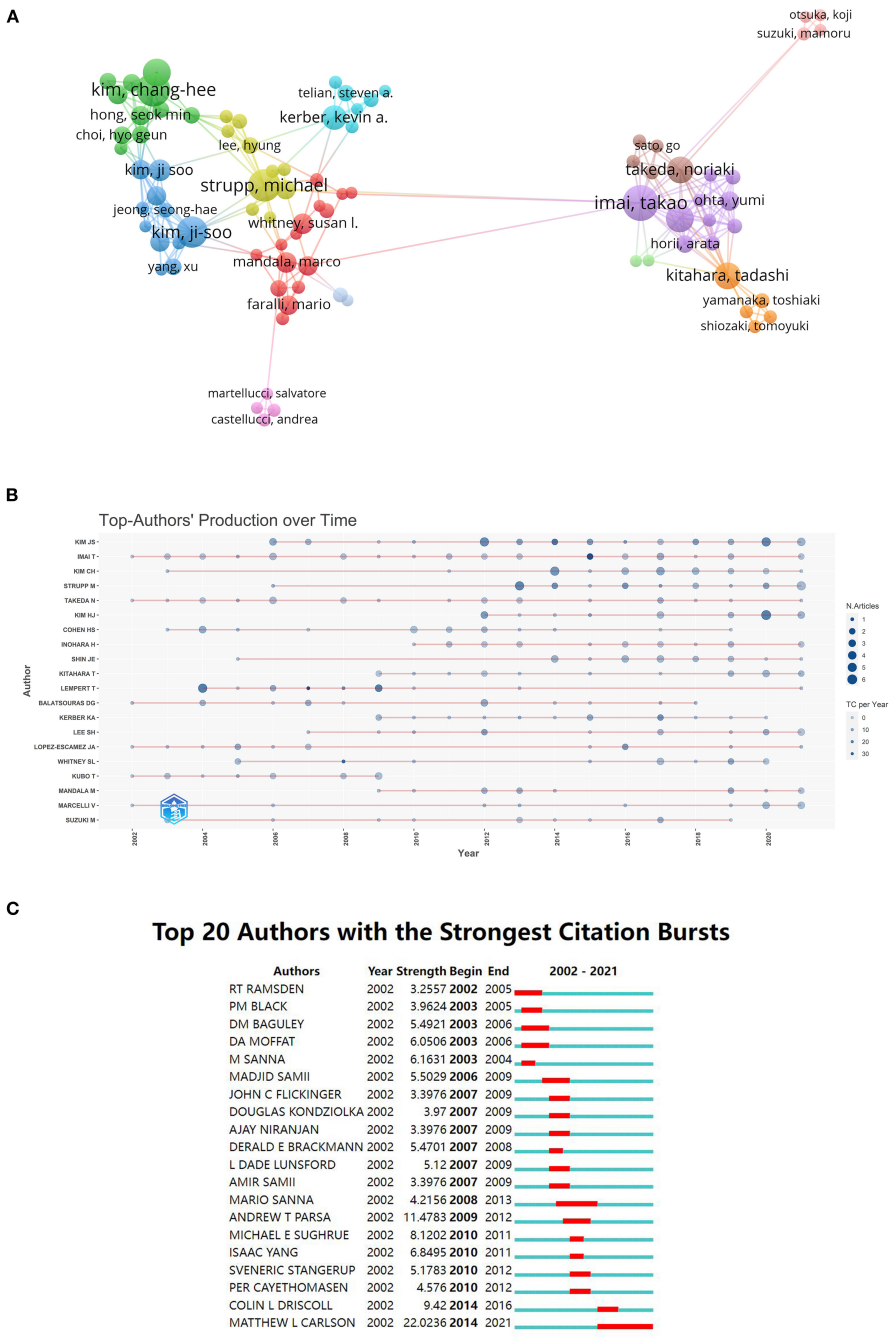
**(A)** A network visualization map of co-authorship in BPPV research from 2002 to 2021. **(B)** The top-20 productive authors in the field over time. **(C)** The top-10 authors with the strongest citation bursts (2002–2021).

A total of 4,594 authors published a considerable amount of research and the 10 most productive authors are presented in Table 3. Authors' collaboration networks are shown in Figure 4A, and the relationship between these authors appears to be fragmented based on the distribution of collaborators. Thus, academic collaborations in this field are comparatively small and insufficient. Figure 4B shows the top-20 productive authors in the field over time. Among the authors, JS Kim of Seoul National University (35 publications; 1,200 citations) contributed the most publications to the field, followed by T Imai of Osaka University (31 publications; 769 citations), and CH Kim of Konkuk University (24 publications; 342 citations), with JS Kim achieving the highest H-index (18). None of the authors had a centrality score that reached 0.01, suggesting there was a relatively low level of cooperation among academic teams from different institutions. The top-10 authors with the largest citation bursts are displayed in Figure 4C.

**Table 3:** The top-10 authors with the most publications related to BPPV.

**Rank**	**Author**	**Count**	**Country**	**Centrality**	**Total number of citations**	**Mean number of citations**	**H-index**
1	Kim JS	35	South Korea	0.00	1,200	34.29	18
2	Imai T	31	Japan	0.00	769	24.74	13
3	Kim CH	24	South Korea	0.00	342	14.25	11
4	Strupp M	23	Germany	0.00	695	30.22	14
5	Takeda N	22	Japan	0.00	439	19.95	12
6	Kim HJ	20	South Korea	0.00	355	17.75	9
7	Shin JE	18	South Korea	0.00	209	11.61	9
8	Cohen HS	17	USA	0.00	402	23.65	12
9	Inohara H	17	Japan	0.00	152	8.94	7
10	Kitahara T	16	Japan	0.00	122	7.63	6

BPPV, benign paroxysmal positional vertigo; USA, United States of America.

### Analysis of keywords

**Figure 5:**
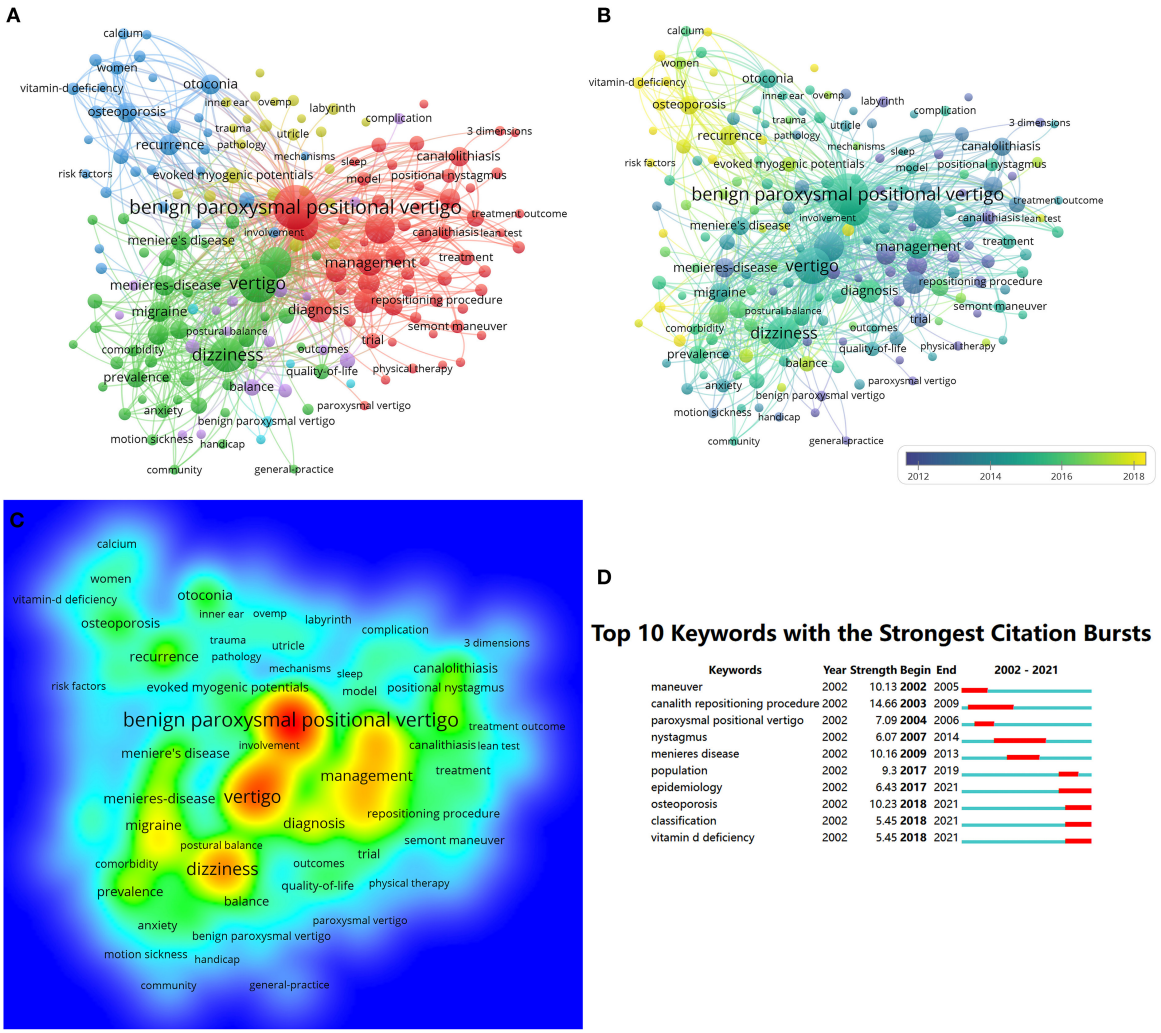
**(A)** A network diagram of 181 keywords classified into five clusters. **(B)** The distribution of keywords is shown in the order of their appearance. **(C)** The density visualization map of the keywords. **(D)** The top-10 keywords with the largest citation bursts (2002–2021).

A total of 3,116 keywords were retrieved, of which 181 occurred >10 times (Figure 5A). The 10 most frequent keywords were BPPV (574), vertigo (403), dizziness (309), nystagmus (212), management (141), diagnosis (133), maneuver (124), canalith repositioning procedure (96), epidemiology (87), and Meniere's disease (84). Five clusters were identified based on their topics, namely, clinical characteristics, pathogenesis, mechanisms, pathological physiology, and treatment of BPPV. Keywords with different (average) years of publication are highlighted in different colors in the time-overlay network map of co-occurring keywords (Figure 5B). Prior to 2012, the majority of research focused on “clinical characteristics,” whereas the newly identified hot spots suggest that current research will focus on “mechanisms” and “management”. The density of the keywords was based on their frequency, as illustrated in the density graph (Figure 5C). We determined that the keywords with the citation bursts were those that were cited frequently during a particular timeframe. Figure 5D shows the top-10 keywords with the largest citation bursts, sorted by time of occurrence. Before 2010, the “canalith repositioning procedure” had the largest burst intensity (14.66), whereas “Meniere's disease” had the largest burst (10.16) in 2010–2016. After 2016, the keyword with the largest citation burst was “osteoporosis” (10.23). It is evident from these changes over time that the focus of research has changed.

### Characteristics of the top-10 co-cited publications

As shown in Table 4, the top-10 publications were ranked by their numbers of co-citations. A total of 7,848 citations for these papers were found, accounting for 10.7% of all the citations (26,496). “Epidemiology of benign paroxysmal positional vertigo: a population-based study,” by Von Brevern M et al. (4), which was published in the journal *Cell* in 2007 was the most frequently cited article (590). Four of the top-10 most cited papers were published in academic journals with an impact factor ≥10 (the Journal of Neurology, Neurosurgery, and Psychiatry, the Canadian Medical Association Journal, Neurology, and the New England Journal of Medicine) and two were published in journals with an impact factor between ≥ 5 and < 10 (Otolaryngology–Head and Neck Surgery and Current Opinion in Neurology).

**Table 4:** The top-10 co-cited publications related to BPPV.

**Rank**	**Title**	**First Author**	**Year**	**Journal**	**Citations**
1	Epidemiology of benign paroxysmal positional vertigo: a population based study	von Brevern M	2007	Journal of Neurology Neurosurgery and Psychiatry	590
2	Clinical practice guideline: Benign paroxysmal positional vertigo	Bhattacharyya N	2008	Otolaryngology–Head and Neck Surgery	402
3	Diagnosis and management of benign paroxysmal positional vertigo (BPPV)	Parnes LS	2003	Canadian Medical Association Journal	298
4	Clinical Practice Guideline: Benign Paroxysmal Positional Vertigo (Update)	Bhattacharyya N	2017	Otolaryngology–Head and Neck Surgery	285
5	Benign paroxysmal positional vertigo: Diagnostic criteria	von Brevern M	2015	Journal of Vestibular Research-Equilibrium & Orientation	262
6	Epidemiology of vertigo	Neuhauser HK	2007	Current Opinion in Neurology	237
7	Practice parameter: Therapies for benign paroxysmal positional vertigo (an evidence-based review)—Report of the Quality Standards Subcommittee of the American Academy of Neurology	Fife TD	2008	Neurology	228
8	Benign Paroxysmal Positional Vertigo	Kim JS	2014	New England Journal of Medicine	199
9	An assessment of gait and balance deficits after traumatic brain injury	Basford JR	2003	Archives of Physical Medicine and Rehabilitation	183
10	Which comes first? Psychogenic dizziness vs. otogenic anxiety	Staab JP	2003	Laryngoscope	161

BPPV, benign paroxysmal positional vertigo.

### Citation analysis

**Figure 6:**
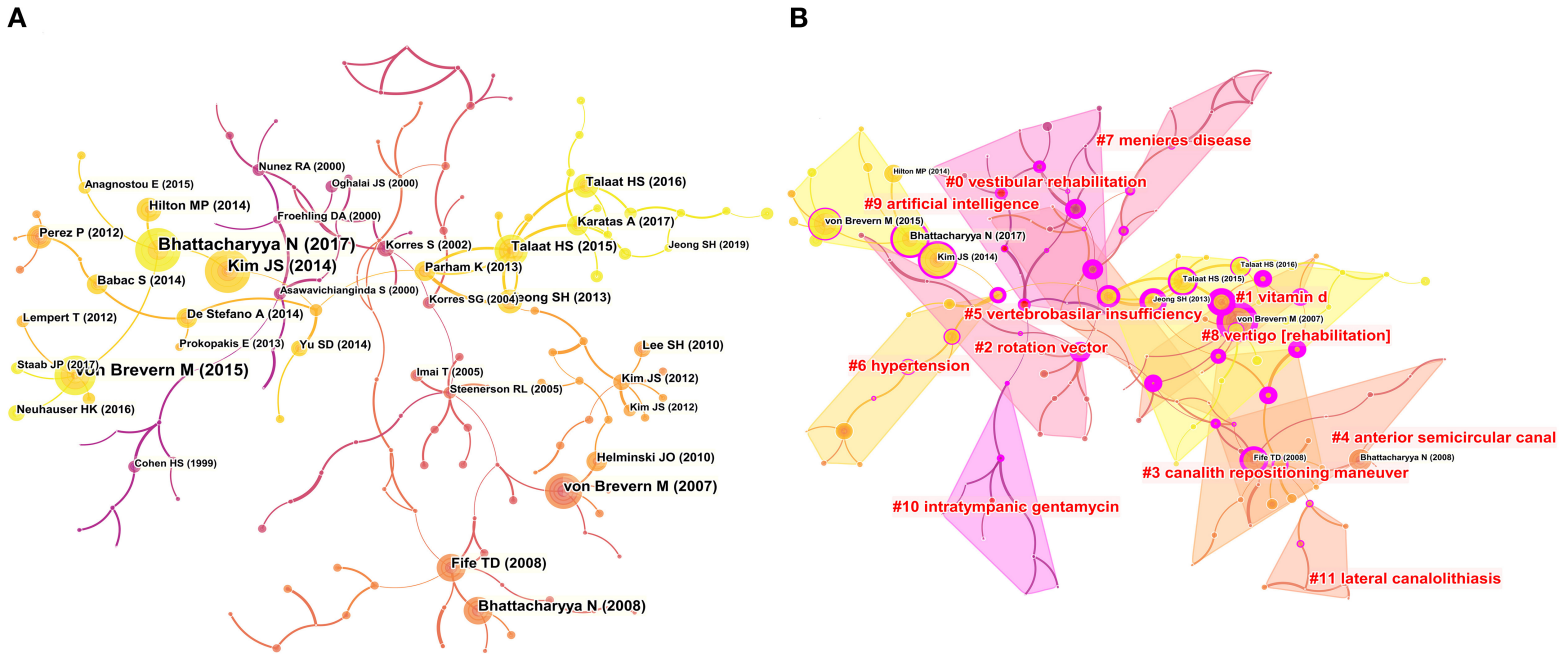
**(A)** Map of co-cited references of publications related to BPPV. **(B)** Cluster analysis of networks with co-cited references.

**Figure 7:**
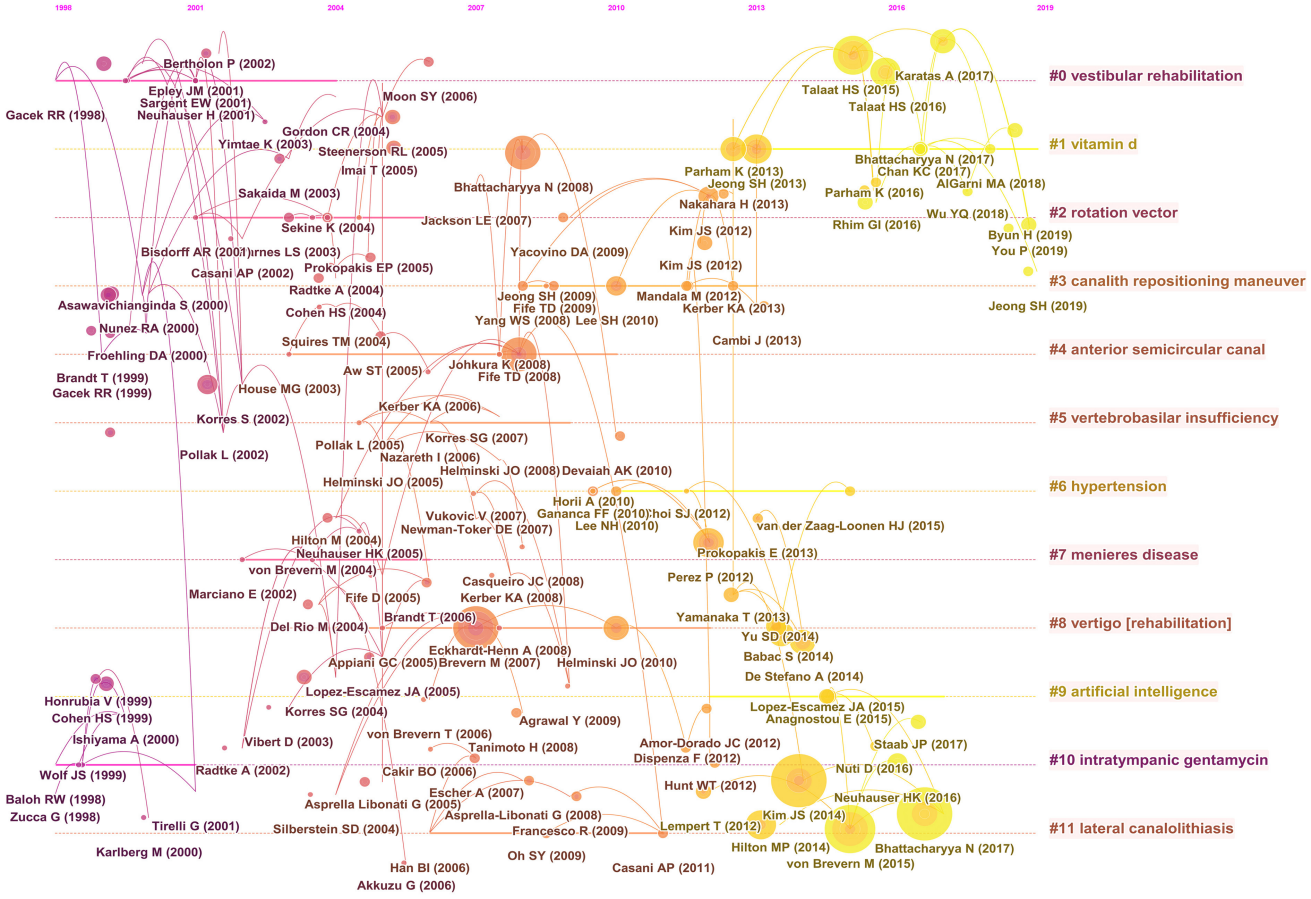
Timelines of the co-cited references with cluster labels.

**Figure 8:**
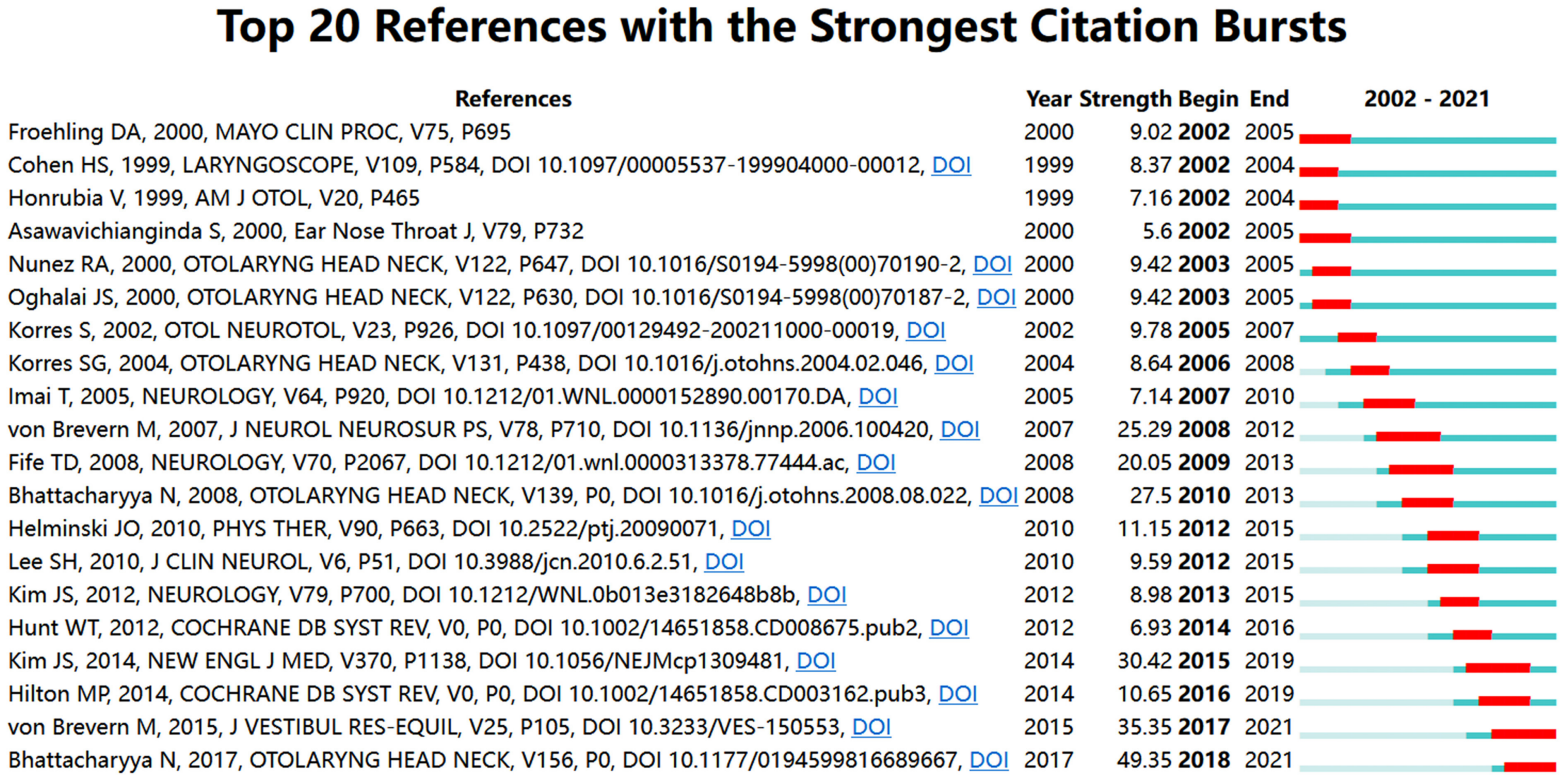
The top-20 references with the largest citation bursts (2002–2021).

Co-cited references are a key indicator in bibliometric analyses. A cluster network map was constructed based on the analysis of 14,975 cited references from 1,419 publications, composed of 151 nodes and 159 links (Figure 6A). Figure 6B illustrates the 10 largest clusters in the reference co-cited network. The largest cluster was “PD” (#0), followed by “vitamin D” (#1), “rotation vector” (#2), “canalith repositioning maneuver” (#3), “anterior semicircular canal” (#4), “vertebrobasilar insufficiency” (#5), “hypertension” (#6), “Meniere's disease” (#7), “vertigo” (8), “artificial intelligence” (9), “intratympanic gentamycin” (10), and “lateral canalolithiasis” (11). Figure 7 plots the top-12 clusters by time in this study, showing the scientific relevance of the references. Figure 8 illustrates emerging research hot spots in relevant areas by highlighting the top-20 references with the largest citation bursts. Given these references, it was apparent that BPPV was a hot topic in otorhinolaryngology and neurology.

### Distribution by journal

**Figure 9:**
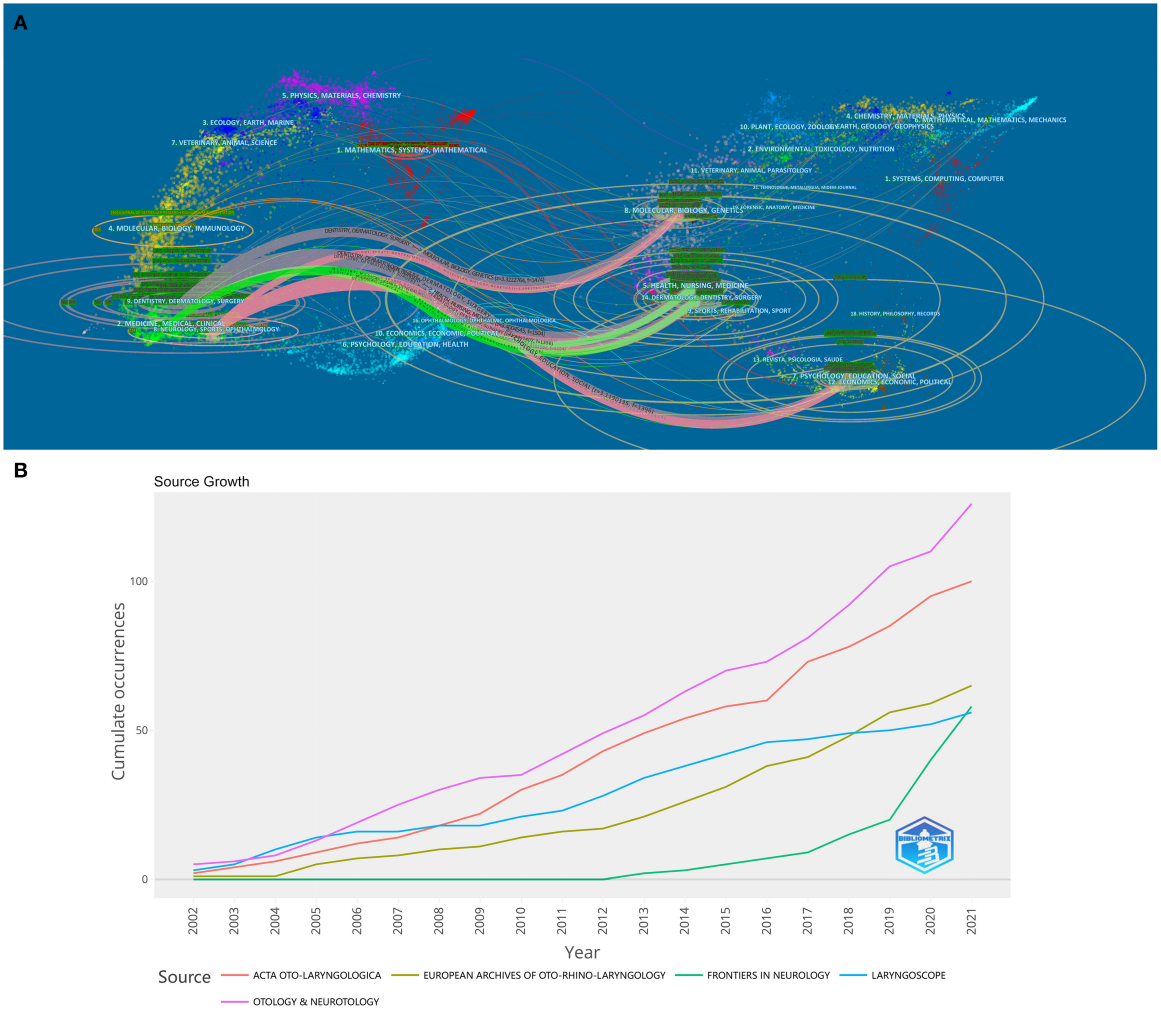
**(A)** A dual-map overlay of journals related to BPPV. **(B)** Cumulative publication trends of the top-5 most prolific journals (2002–2021).

Figure 9A shows a dual-map overlay of journals. The left side represents the studies published in journals, and the right side represents the journals in which those published studies were cited, with routes indicating the citation relationships between the journals. Four gray, two greens, and two pink paths represent the citation paths in this diagram. The gray routes indicate the papers published in dentistry, dermatology, and surgery journals that were cited in molecular, biology, and genetics; dermatology, dentistry, and surgery; and health, nursing, and medicine journals. The green paths indicate studies published in medicine, medical, and clinical journals that were frequently cited in health, nursing, medicine and dermatology, dentistry, and surgery journals. The pink paths indicate the studies published in molecular, biology, and genetics journals, which were often cited in articles published in neurology, sports, and ophthalmology journals.

The cumulative publication trends of the top-5 most prolific journals are shown in Figure 9B. Table 5 presents the ten most prolific journals, which published 44.2% (627 publications) of the total publications; 10% of them were ranked in the first quartile (Q1). It is noteworthy that two of these journals published more than 100 papers. Otology & Neurotology, the European Archives of Oto-Rhino-Laryngology, and Acta Oto-Laryngologica published the most articles related to BPPV (total = 293 articles, 20.6%), with Otology & Neurotology having the largest number of articles (126) and the highest H-index (28). However, Otolaryngology-Head and Neck Surgery had the highest impact factor and mean number of citations. Q1 of the Journal Citation Report included Otolaryngology—Head and Neck Surgery; Q2 included Frontiers in Neurology, the American Journal of Otolaryngology, and the European Archives of Oto-Rhino-Laryngology, and Q3 consisted of Otology & Neurotology, Laryngoscope, the Journal of Laryngology and Otology, and Auris Nasus Larynx.

**Table 5:** The top-10 most prolific journals for publications related to BPPV.

**Rank**	**Journal**	**Count**	**Country**	**Journal citation reports (2021)**	**Impact factors (2021)**	**Total number of citations**	**Mean number of citations**	**H-index**
1	Otology & Neurotology	126	USA	Q3	2.619	2,254	17.89	28
2	Acta Oto-Laryngologica	101	Norway	Q4	1.698	1,203	11.91	20
3	European Archives of Oto-Rhino-Laryngology	66	Germany	Q2	3.236	1,026	15.55	18
4	Laryngoscope	59	USA	Q3	2.970	1,641	27.81	24
5	Frontiers in Neurology	58	Switzerland	Q2	4.086	378	6.52	11
6	Otolaryngology-Head and Neck Surgery	54	USA	Q1	5.591	1,814	33.59	22
7	American Journal of Otolaryngology	42	USA	Q2	2.873	492	11.71	15
8	Journal of Laryngology and Otology	42	UK	Q3	2.187	413	9.83	13
9	Journal of Vestibular Research-Equilibrium & Orientation	40	USA	Q4	2.354	736	18.40	14
10	Auris Nasus Larynx	39	Netherlands	Q3	2.119	528	13.54	12

BPPV, benign paroxysmal positional vertigo; USA, United States of America.

## Discussion

Although treatment methods for BPPV have advanced, many patients experience recurrences after therapy (2). The impact of the higher prevalence of BPPV on health care and society is enormous. The medical, administrative, and indirect costs of BPPV should not be overlooked. As stated earlier, the cost of reaching a BPPV diagnosis has been estimated to be ~$2000, and some individuals also experience disruptions in their daily lives and loss of work due to BPPV (17). The global healthcare cost related to BPPV diagnoses is closer to $2 billion annually. At present, BPPV is receiving growing attention worldwide, and related studies are increasing annually. Thus, a comprehensive overview of the current global trends in BPPV research is especially important. This study analyzed the bibliometric characteristics of global publications in the field of BPPV and revealed the main research hot spots and trends from 2002 to 2021. Over the past 20 years, the total number of BPPV articles has increased consistently, demonstrating that research on this illness is taken seriously by researchers and clinicians. All as important etiologies of vestibular peripheral vertigo, Meniere's disease, vestibular neuritis and BPPV, show some similarities in their clinical features. To better observe other areas within the specialty, we also used the same method to count relevant publications from Meniere's disease and vestibular neuritis (Supplementary Figure S1). As can be observed by the growth curves, the most marked trend in the growth of publications related to BPPV over the past two decades is followed by Meniere's disease. And the trend in publications regarding vestibular neuritis is not obvious. We predict that this topic of BPPV will continue to be a popular focus of searches in the next decade, and that the number of related publications will continue to grow.

The USA takes the leading position for contributing more than 20% of the global publications on BPPV. The majority of prolific institutions are located in developed countries, which has contributed to the progress of BPPV-related research. This trend indicates a mature research environment and a large investment in research by these countries or regions that reflect an urgent need for the effective treatment of BPPV. The size of their population and the economic differences between countries may have a significant effect on the number of publications. The USA is the most frequent collaborator with other countries, ranking first on centrality scores. Korea, China, and Italy published numerous articles; however, their centrality scores indicated that they rarely collaborated internationally. Countries with low centrality scores can increase their international communication and cooperation, while building friendly, cooperative partnerships, especially with colleagues using current technology in this field. As seen in the centrality scores, institutions of higher education, such as Johns Hopkins, Harvard, and Michigan collaborate most frequently with each other, which contributes significantly to the development of their disciplines. JS Kim, T Imai, and CH Kim were the top-3 most productive authors. There is a marked geographical distribution of related authors. Most of these scholars come from developed countries and are employed by departments of neurology or otorhinolaryngology at university hospitals.

The journals with the most BPPV-related publications were Otology & Neurotology, Acta Oto-Laryngologica, the European Archives of Oto-Rhino-Laryngology, Laryngoscope, and Frontiers in Neurology. These journals are well known in the field of otolaryngology and neurology, suggesting that BPPV is a hot topic in otolaryngology and neurology. The most co-cited articles during 2002–2021 suggest a strong academic interest in the clinical aspects of BPPV. Notably, “Clinical Practice Guidelines. Benign Paroxysmal Positional Vertigo” (18) is acknowledged as the guideline for the treatment of BPPV by clinicians worldwide. Bhattacharyya et al. (19) published the 2008 version as an update of this guideline. Compared to the previous guideline, the new version includes a recommendation for canalith repositioning post-procedural restrictions, expands recommendations related to vestibular testing and radiological examinations, emphasizes the application of audiometric testing, and adds visual algorithms for diagnosis and treatment. The dual-map overlay presents a macro-perspective of the changes in the research content from the disciplinary perspective, and shows the distribution of journals. As seen in the diagram, surgery and clinical medicine are the core disciplines associated with BPPV. The three main pathways in the visualization diagram show that BPPV research has begun to shift from a single discipline to a multidisciplinary perspective.

The analysis of high-frequency keyword clustering indicates that the pathophysiological mechanisms of BPPV and its therapeutic approaches and efficacy assessments remain hot topics in the field. The research focus on BPPV was observed at various times over 20 years: “maneuver” (2002–2005), “canalith repositioning procedure” (2003–2009), “Meniere's disease” (2009–2013), “population” (2017–2019), and “osteoporosis” (2018–2021). Osteoporosis is a common metabolic disease, mainly caused by an imbalance in the bone's conversion rate, resulting in less bone formation than bone resorption (20). Multiple bone turnover parameters, including bone mass, vitamin D, paracrine hormones, and other bone metabolites, are likely to be abnormal in patients with osteoporosis. Such changes in the metabolism of calcium ions and associated hormones are thought to affect the functioning of various organs, including homeostasis of the inner ear related to otolithic function (21). Otoconia are primarily composed of calcium carbonate crystals; therefore, some studies have suggested that osteoporosis is associated with BPPV. A case control study reported that patients with BPPV were 1.29 times more likely to be diagnosed with osteoporosis than were patients without BPPV (22). Another finding indicated that patients with osteoporosis had a 1.82 times higher prevalence of BPPV than those without osteoporosis (23). A study from Japan also reported that patients with osteoporosis had a significantly higher recurrence rate of BPPV (56.3%) than those with normal bone mineral density (16.2%) (24). The association between BPPV and osteoporosis is a popular topic, but the number of relevant studies is small. Hence, more clinical data are needed to further examine this association.

The timeline of the clustering diagram shows that vitamin D in BPVV has become a frontier of research in recent years. Vitamin D is a factor known to be associated with bone metabolism, as well as a critical element of calcium metabolism (25). The stages of otolith formation, development, and degeneration occur due to active metabolism of calcium ions within the vestibular apparatus, and disturbances in calcium metabolism in osteoporosis have been reported to cause BPPV (26). Vitamin D promotes the expression of several calcium binding proteins and receptors in the inner ear (27). Changes in the levels of hydrogen and the calcium ion in the endolymph can cause a reduction in otolith size and malformation (28). Thus, vitamin D deficiency could influence calcium metabolism in the inner ear, resulting in the progression of BPPV. The findings of several studies on calcium carbonate and glycoprotein metabolism suggest that vitamin D participates in the process of calcium metabolism, and vitamin D insufficiency promotes the formation of BPPV (29). A study from Egypt showed that vitamin D concentrations in the control group were significantly different from the concentrations found in the non-recurrent and recurrent BPPV groups (19.5 vs. 16.0 vs. 11.9 ng/mL) (26). Several studies have confirmed that the administration of vitamin D supplementation decreases the rate of disease recurrence among patients with BPPV and vitamin D-deficiency. A study from Australia reported a decrease in relapse rates after vitamin D supplementation was administered to four patients with vitamin D deficiency who had a long history of recurrent severe BPPV (30). Another study found that after 3 months of vitamin D supplementation in 28 vitamin D-deficient patients with BPPV, the recurrence rate was significantly lower compared to the control group during the 18-month follow-up period (31). The use of vitamin D-containing drugs might reduce the severity and recurrence of BPPV in patients who are also vitamin D-deficient. However, further prospective studies with larger sample sizes are necessary to clarify this issue.

Currently, treatment of all forms of BPPV is mainly through canal repositioning maneuvers as a way of releasing the canal from lithiasis. This maneuver is very effective, with a 98% success rate after three sessions (32). In addition, spontaneous recovery is common and usually occurs 6 to 8 weeks after the initial appearance of symptoms. Therefore, BPPV is usually regarded as a benign disease. However, a small proportion of patients still suffer from highly disturbing levels and frequency of BPPV attacks even after repeated spinal repositioning operations. Long-term disabling BPPV is defined as refractory vertigo that persists for up to 1 year after physical therapy. Some of patients with BPPV may have their social, occupational and family activities affected and may be considered handicapped. For these patients, surgery may be considered and various techniques have been suggested, including microvascular decompression, utricular ablation, singular nerve section, and posterior semicircular canal occlusion (33). Both semicircular canal occlusion and singular nerve section are effective surgical options for patients with difficult-to-control, incapacitating BPPV. Although semicircular canal occlusion needs a retroauricular, transmastoid approach, it is an easier and safer procedure, and should be regarded as the best option (34).

### Strengths and limitations

The publications included in this study covered most of the BPPV-related research literature over the past 20 years. This bibliometric analysis was relatively objective and comprehensive, clarifying the previous and current status of research on BPPV, and predicting future research directions. It should be noted that this research has several limitations. First, given the authority and comprehensiveness of the Web of Science Core Collection, we did not search any other important databases, such as MEDLINE, Scopus and Embase. Thus, our data might not be representative of all available publications. Second, the results of the bibliometric analysis might differ from the results of real-time studies due to the continuous updating of the database. Several papers published recently with a potential impact might not have appeared in the present study because they were cited less often in other studies. Third, only English-language publications were included in this study; therefore, some bias might exist. The final limitation is that the per capita situation was not considered when comparing countries with different population sizes. Bibliometrics is a quantitative analysis of academic publications, and high citations do not necessarily represent high quality (35). For instance, highly cited publications do not always meet clinical needs and sometimes may not even be clinically relevant, especially for basic medical research that focuses on a single molecule or a signaling pathway. Finally, recently published high-quality articles may not be included in our analysis because of low citations. In the future, we may use multiple methods combined with specific literature assessments to obtain a deeper understanding of this research area. Despite these limitations, we believe this study presents a global perspective of the last 20 years of BPPV research for researchers worldwide.

To the best of our knowledge, this study is the first to analyze trends and hot spots systematically in BPPV research over the past 20 years using a bibliometric approach. This study can guide academicians choosing proper research directions and facilitate their recognition of trends and frontiers in the field. Global scholars can be expected to report high quality clinical evidence related to BPPV in the near future. Deep and extensive collaboration between countries, regions, institutions, and authors can be expected to facilitate the development of more effective treatments for BPPV, which will ultimately benefit patients.

## Data availability statement

The raw data supporting the conclusions of this article will be made available by the authors, without undue reservation.

## Author contributions

FZ, BY, and GY conceived the study. FZ, TZ, and JL collected the data. TZ and YM re-examined the data. FZ and JL analyzed the data. FZ wrote the manuscript. BY, TZ, and GY reviewed and revised the manuscript. All authors contributed to the article and approved the submitted version.

## Funding

This work was supported by the Science and Technology Foundation of Guizhou Province (D2011160), National Natural Science Foundation Cultivation Project of Affiliated Hospital of Guizhou Medical University (20NSP023), and Science and Technology Fund Project of Guizhou Provincial Health Commission (gzwkj 2021-328).

## Conflict of interest

The authors declare that the research was conducted in the absence of any commercial or financial relationships that could be construed as a potential conflict of interest.

## Publisher's note

All claims expressed in this article are solely those of the authors and do not necessarily represent those of their affiliated organizations, or those of the publisher, the editors and the reviewers. Any product that may be evaluated in this article, or claim that may be made by its manufacturer, is not guaranteed or endorsed by the publisher.

## Footnotes

^1^http://www.bibliometrix.org.

^2^https://bibliometric.com.

## Abbreviations

BPPV: benign paroxysmal positional vertigo
USA: United States of America.
