# Supplementary figures and images for: Global trends in the research on benign paroxysmal positional vertigo: A 20-year bibliometric and visualization analysis
Source: Front Neurol. 2022 Oct 17;13:1046257. doi: 10.3389/fneur.2022.1046257 (PMC9618818; doi:10.3389/fneur.2022.1046257)

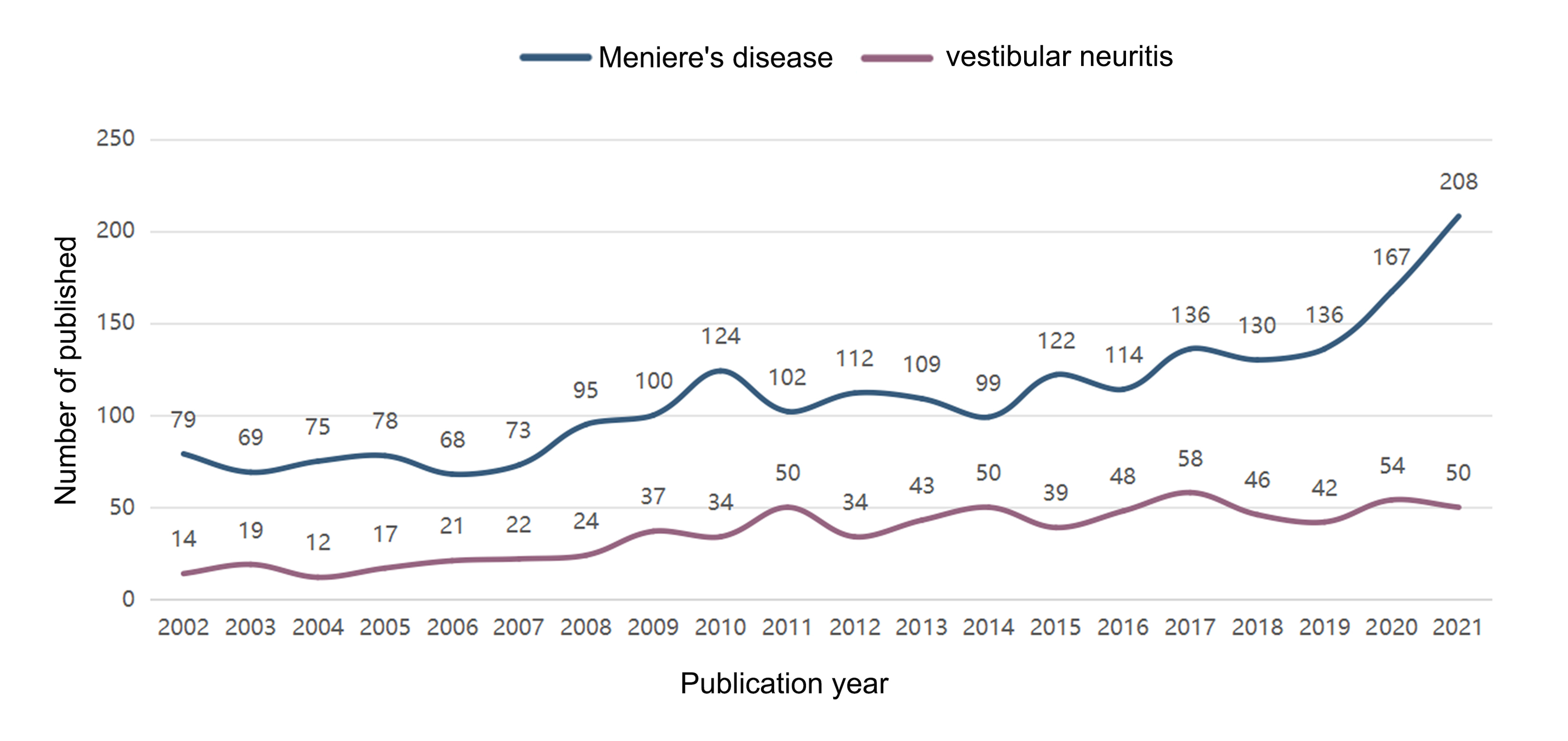

Supplement: Supplementary file 1 [file Image_1.JPEG]
